# Production, Characterization and Antioxidant Potential of Protease from *Streptomyces* sp. MAB18 Using Poultry Wastes

**DOI:** 10.1155/2013/496586

**Published:** 2013-08-07

**Authors:** Panchanathan Manivasagan, Jayachandran Venkatesan, Kannan Sivakumar, Se-Kwon Kim

**Affiliations:** ^1^Marine Biotechnology Laboratory, Department of Chemistry, Pukyong National University, Busan 608-737, Republic of Korea; ^2^Centre of Advanced Study in Marine Biology, Faculty of Marine Sciences, Annamalai University, Parangipettai, Tamil Nadu 608 502, India; ^3^Marine Bioprocess Research Center, Pukyong National University, Busan 608-737, Republic of Korea

## Abstract

Poultry waste is an abundant renewable source for the recovery of several value-added metabolites with potential industrial applications. This study describes the production of protease on poultry waste, with the subsequent use of the same poultry waste for the extraction of antioxidants. An extracellular protease-producing strain was isolated from Cuddalore coast, India, and identified as *Streptomyces* sp. MAB18. Its protease was purified 17.13-fold with 21.62% yield with a specific activity of 2398.36 U/mg and the molecular weight was estimated as 43 kDa. The enzyme was optimally active at pH 8–10 and temperature 50–60°C and it was most stable up to pH 12 and 6–12% of NaCl concentration. The enzyme activity was reduced when treated with Hg^2+^, Pb^2+^, and SDS and stimulated by Fe^2+^, Mg^2+^, Triton X-100, DMSO (dimethyl sulfoxide), sodium sulphite, and **β**-mercaptoethanol. Furthermore, the antioxidant activities of protease were evaluated using *in vitro* antioxidant assays, such as DPPH radical-scavenging activity, O_2_ scavenging activity, NO scavenging activity, Fe^2+^ chelating activity, and reducing power. The enzyme showed important antioxidant potential with an IC_50_ value of 78 ± 0.28 mg/mL. Results of the present study indicate that the poultry waste-derived protease may be useful as supplementary protein and antioxidant in the animal feed formulations.

## 1. Introduction 

Feather is composed of over 90% protein, the main component being keratin, a fibrous and insoluble protein highly cross-linked with disulphide and other bonds. In mature chicken, feather accounts up to 5–7% of the live weight. Worldwide, several million tons of feather is generated annually as waste by poultry-processing industries. Considering its high protein content, this waste could serve as a good source of protein and amino acids for animal feed and for many other applications. However, because of the insoluble nature of keratin and its resistance to enzymatic digestion by animal, plant, and many known microbial proteases, use of feather as a source of value-added products has been very limited.

Thermophilic actinobacteria produce many degradative enzymes [1] and can play a major role in the biodegradation of keratinaceous waste materials [2]. Biodegradation of feathers by microorganisms represents a method for improving the utilization of feathers as a feed protein [3] and amino acids as pure chemicals [4]. Feather may also find an important application in the fermentation industry for the production of commercial enzymes.

Several studies have been made on the proteolytic enzymes of mesophilic actinobacteria [5]. In contrast, relatively little work of a similar nature has been published on alkaline protease-producing actinobacteria. In the present study, an attempt has been made to optimize the culture conditions of *Streptomyces* sp. MAB18 for protease production using poultry wastes. In addition, protease from *Streptomyces* sp. MAB18 was purified and characterized, and the antioxidant activity of the culture supernatant was analyzed.

## 2. Material and Methods

### 2.1. Materials

Chicken feathers (whole feather) were collected immediately after slaughtering of the chickens and extensively washed with tap water until the effluent became very clear and finally with distilled water. The washed feathers were dried under sunlight and then further dried at 60°C for 48 h. After drying, the large feather stocks were cut by hand into smaller pieces to fit to the culture flask. They were stored at 4°C until used [6]. Standard proteins and tyrosine were purchased from Sigma-Aldrich, India. Other reagents were from Merck (Germany). All other chemicals and bacteriological media were from standard sources.

### 2.2. Isolation and Screening of Marine Actinobacteria

A marine actinobacterium *Streptomyces* sp. MAB18 was isolated from the marine sediments of Cuddalore coast (lat 11°42′ N, long 79°52′ E), India, and screened for protease production on gelatin agar medium (gelatin, 10 g; peptone, 5 g; beef extract, 5.0 g; agar, 20.0 g; and pH 8.0), and incubated at 50°C. After incubation, clear zones developed around the colony were considered positive for protease activity. The selected strain was grown in liquid medium prepared as above but in which gelatin was substituted by 10 g/L chicken feather. The cultures were incubated at 50°C with rotary shaking and solubilisation of the feather was observed visually. The level of protease production was checked from the culture supernatant obtained after centrifugation [7].

### 2.3. Taxonomic Studies and 16S rDNA Sequencing

The selected strain was identified according to Bergey's Manual of Determinative Bacteriology (1974) and the keys proposed [8].

A molecular identification of the isolate was achieved by 16S rDNA sequencing. DNA extraction was performed by the CTAB method [9]. The primer sequences were chosen from the conserved regions previously reported for the bacterial 16S rDNA [10]. Sequencing was done using forward primer (F 5′-CAGGCCTAACACATGCAAGTC-3′) and reverse primer (R 5′-GGGCGGTGTGTACAAGGC-3′). PCR reactions were performed with the following program for the 16S rRNA gene: 30 cycles consisting of 95°C for 1 min, 55°C for 1 min, and 72°C for 1.5 min, followed by a final extension step of 5 min at 72°C. The 16S rDNA sequence was analyzed by an automated DNA sequencer (Applied Biosystems). The sequence was analyzed for homology using CLUSTAL X package [11].

### 2.4. Protease Production

Whole-feather medium (WFM), which contained whole-chicken feather (WCF) 10 g, peptone 5 g, beef extract 5 g, K_2_HPO_4_ 1 g, MgSO_4_·7H_2_O 0.5 g, CaCl_2_ 0.5 g, Na_2_CO_3_ 5 g, NaCl 5 g, and pH 8.0 was used for protease production. Medium (100 mL) was dispensed in 500 mL Erlenmeyer flask and sterilized at 110°C for 20 min. Each flask was inoculated with 1 mL of 48-hours-old seed culture (8 × 10^9^ CFU/mL), prepared in the same medium, and incubated at 55°C, 200 rpm for 7 day. Culture broth was centrifuged at 10000 ×g for 10 min and the supernatant was used as an enzyme [12].

### 2.5. Enzyme Assays

#### 2.5.1. Protease Activity

Protease activity was determined with a modification of the method described by Cheng et al. [13]. Reaction mixture (2 mL) containing 1 mL of casein 1% (w/v) (dissolved in 25 mM glycine NaOH buffer pH (10.0)) and 0.95 mL of glycine NaOH buffer was preincubated at 50°C. The reaction was initiated by the addition of 0.05 mL of suitably diluted enzyme solution and kept at 50°C for 20 min reaction; 2 mL of trichloroacetic acid 10% (w/v) was added to terminate the reaction and the mixture was allowed to stand at room temperature for 1 h. The reaction mixture was centrifuged at 10000 ×g for 10 min and the absorbance of the supernatant was determined at 280 nm. Protease (1 U) activity was defined on the amount of enzyme required to liberate 1 *μ*g of tyrosine per minute under experimental conditions [14].

#### 2.5.2. Keratinase Activity

Keratinase activity was assayed by the modified method of Plackett and Burman [15]. The mixture of 10 mg of feather powder suspended in 1 mL of 50 mM Tris-HCl buffer (pH 7.5) containing 1 mM CaCl_2_ and 1 mL of culture filtrate was incubated at 35°C with shaking at 125 rpm for 15 min in a water bath shaker. This elevated temperature was used for the enzyme incubation to accelerate substrate hydrolysis. The enzyme reaction was terminated by adding 2 mL of trichloroacetic acid (TCA) solution (0.11 M trichloroacetic acid, 0.22 M sodium acetate, and 0.33 M acetic acid) into the reaction mixture. The mixture was then centrifuged at 10 000 ×g, 4°C for 30 min and the absorbance of the supernatant was spectrophotometrically measured at the wavelength of 275 nm (UV-1800, Shimadzu scientific instruments, USA). The enzyme inactivated by TCA solution was used as a control. One unit (U) of keratinase activity has been expressed as 1 *μ*mol of tyrosine released per minute under the specific conditions [13].

### 2.6. Optimization of Medium Components

Medium optimization was carried out by statistical approaches. Physical parameters such as pH, agitation, and nutritional parameters, that is, carbon and nitrogen sources, were first standardized by one-variable-at-a-time method. Following this, the Plackett-Burman (PB) design and response surface methodology (RSM) were used to optimize the medium composition for maximum protease production.

#### 2.6.1. Selection of the Most Suitable Carbon and Nitrogen Sources by One-Variable-at-a-Time Approach

Initial screening of the most significant carbon and nitrogen sources allowing the maximum protease production was performed by the one-variable at-a-time approach. To check the effect of various carbon and nitrogen sources on protease production, media were supplemented with 1% (w/v) of different carbon and nitrogen sources. The flasks were inoculated with 2% inoculum and incubated in a shaking incubator with a shaking speed of 180 rpm at 55°C for 7 days. Samples were collected every 12 h and assayed for growth as well as enzyme production.

#### 2.6.2. Plackett-Burman Design (PB Design)

Important medium components with respect to their main effects were screened by the Plackett-Burman design with a two-factorial design. It identifies the main physico-chemical parameters required for maximal protease production by screening *n* variables in *n* + 1 experiments; each variable was examined at two levels [15]. Table S1A see (Supplementary Matrial available online at http://dx.doi.org/10.1155/2013/496586) lists the factors under investigation as well as the levels of each factor used in the experimental design with the symbol code and actual level of the variables. “Design expert software” (Minitab package version 16.0) was used to analyse the experimental Plackett-Burman design.

#### 2.6.3. Optimization of Key Ingredients by Central Composite Design (CCD)

Levels of four significant factors and the interaction effects between various medium constituents which influence the protease production significantly were analysed and optimized by the response surface methodology, using a CCD design. The significant factors utilized were whole-chicken feather (WCF), peptone, NaCl, and Na_2_CO_3_. In this study, the experimental plan consisted of 27 trials and the independent variables were studied at three different levels: low, middle, and high as shown in Table S2A. All the experiments were done in duplicate and the average protease production obtained was taken as the dependent variable or response (*Y*).

#### 2.6.4. Statistical Analysis and Modeling

Data obtained from RSM on protease production were subjected to analysis of variance (ANOVA). The experimental results of RSM were fitted via the response surface regression procedure, using the following second-order polynomial equation:

(1) $$\begin{array}{c} Y=\beta_{0}+\sum_{i}^{}\beta_{i}X_{i}+\sum_{\;ii\;}^{}\beta_{ii}X_{i}^{2}+\sum_{\;ij}^{}\beta_{ij}X_{i}X_{j}, \end{array}$$

where *Y*_*i*_ is the predicted response, *X*_*i*_ *X*_*j*_ are independent variables, *β*_0_ is the offset term, *β*_*i*_ is the *i*th linear coefficient, *β*_*ii*_ is the *i*th quadratic coefficient, and *β*_*ij*_ is the *ij*th interaction coefficient. However, in this study, the independent variables were coded as *X*_1_, *X*_2_, *X*_3_, and *X*_4_. Thus, the second-order polynomial equation can be presented as follows:

(2) Y=β0+β1X1+β2X2+β3X3+β4X4+β11X12+β22X22+β33X32  +β44X42+β12  X1X2+β13X1X3+β14X1X4+β23X2X3+β24X2X4+β34X3X4.

The statistical software package Minitab package version 16.0 was used for the regression analysis of the experimental data and also to plot the response surface graphs. The statistical significance of the model equation and the model terms was evaluated via Fisher's test. The quality of fit the second-order polynomial model equation was expressed via the coefficient of determination (*R* ^2^) and the adjusted *R* ^2^. The fitted polynomial equation was then expressed in the form of three-dimensional surface plots, in order to illustrate the relationship between the responses and the experimental levels of each of the variables utilized in this study. The point optimization method was employed in order to optimize the level of each variable for maximum response. The combination of different optimized variables, which yielded the maximum response, was determined in an attempt to verify the validity of the model.

### 2.7. Time Course of Protease Production

The kinetics of protease production were followed in batch cultures at optimum conditions. The experiment was designed for 7 days starting from the log phase to stationary phase under submerged culture conditions. The resultant cell free supernatant was removed by filtration followed by cold centrifugation at 10 000 ×g at 4°C for 20 min. The supernatant was analyzed for protease production.

### 2.8. Purification of Protease Enzyme

For purification of protease, ammonium sulphate was added to the culture supernatant to obtain 60% saturation (w/v) and allowed to stand overnight at 4°C. The precipitate collected through centrifugation at 10 000 ×g for 15 min was dissolved in 50 mM Tris-HCl buffer (pH-8) and dialyzed against the same buffer (4°C). The dialysate was loaded on DEAE-Cellulose column (5 × 25 cm) and eluted with linear gradient of NaCl (0–1.0 M) at a flow rate of 0.5 mL/min. Fractions were collected and assayed for enzyme activity and fractions which exhibited enzyme activity were pooled together and concentrated by ammonium sulphate precipitation. The resultant precipitate was collected by centrifugation and dissolved in 50 mM Tris-HCl buffer (pH-8.0). Concentrated fractions were loaded onto a Sephadex G-50 column (2.5 × 25 cm) equilibrated with 50 mM Tris-HCl buffer (pH-8) and eluted with the same buffer at a flow rate of 15 mL/h. Fractions exhibiting protease activity were pooled together and used as a purified enzyme for further characterization study.

### 2.9. Determination of Protein

Protein concentration of the protease in supernatant was determined by the method, using bovine serum albumin as the standard [16].

### 2.10. Molecular Weight Determination in SDS-PAGE

SDS-PAGE (10%) was performed as described, under reducing conditions. The molecular weight was determined by interpolation from a linear semilogarithmic plot of relative molecular weight versus the relative mobility, using broad-range standard molecular weight markers (29, 43, 66, 97, and 200 kDa) [17].

### 2.11. Effect of pH on Activity and Stability of Protease

The optimum pH of alkaline protease was determined with casein 1% (w/v) as substrate dissolved in different buffers (citrate phosphate, pH 5-6, sodium phosphate, pH 7.0, Tris-HCl, pH 8.0, and glycine NaOH, pH 9–13). The pH stability of alkaline protease was determined by preincubating enzyme in different buffers for 10 h at 50°C. 

### 2.12. Effect of Temperature and NaCl on the Enzyme Activity and Stability

Effect of temperature on the enzyme activity was determined by incubating the reaction mixture (enzyme + substrate) at different temperatures (30–75°C). To determine the temperature stability, the purified enzyme was preincubated at different temperatures (30–70°C), for 1 h and then residual activity (%) was assayed under standard assay conditions. Effect of NaCl on the enzyme activity was studied by varying the concentrations of NaCl% (w/v). Enzyme activity has been expressed as percentage relative activity.

### 2.13. Effect of Inhibitors and Surfactants

The effect of inhibitors and surfactants on enzyme activity took place under standard enzyme assay conditions where the assay cocktail was supplemented with phenylmethylsulfonyl fluoride (PMSF), (10 mM), EDTA (1 mM), cystine (1 mM), SDS (0.1%), Tween-80 (0.1%), and Triton X-100 (0.1%). The effect was assessed by comparing with the control.

### 2.14. Effect of Metallic Salts and Inhibitors on Protease Activity

The enzyme was preincubated for 1 h in concentrations (1, 3, 5, and 10 mM) of different metallic salts (FeCl_2_, HgCl_2_, MgCl_2_, AgNO_3_, CuCl_2_, PbCl_2_, CaCl_2_, NiCl_2_, and MnCl_2_) and then the residual activity (%) was measured under optimum conditions. From this, the inhibitory effect of various compounds on the enzyme and the nature of the alkaline protease were determined.

### 2.15. Detergent Stability

Stability of the protease in commercial detergents were tested by incubating measured quantity of the enzyme (500 *μ*L) with the solutions of the different commercial detergents at a detergent concentration of 7 mg/mL (to simulate washing conditions) [18] for 1 h. The detergents tested were Ariel, Tide (Procter and Gamble Ltd.), Rin, Surf excel (Hindustan Lever Ltd.), and Henko (SPIC India Ltd.), which are widely used in India. Suitable aliquots were withdrawn at different time intervals (at 15, 30 and 60 min), for 1 h, and the residual activity was measured by standard assay procedure and compared with the control (incubated under similar conditions, without any detergent) and the relative activity has been expressed in % taking the value given by control as 100%. Also the detergent solutions in the same concentration used for this study, but without incubating with the enzyme, were assayed for protease activity to rule out the possibility of any protease (if at all present) contained as ingredient of the detergent [19, 20].

### 2.16. Antioxidant Activity

#### 2.16.1. DPPH Radical-Scavenging Assay

DPPH radical-scavenging activity of the hydrolysates was determined as described [21]. A volume of 500 *μ*L of each sample at different concentrations was mixed with 500 *μ*L of 99.5% ethanol and 125 *μ*L of 0.02% DPPH in 99.5% ethanol. The mixture was then kept at room temperature in the dark for 60 min, and the reduction of DPPH radical was measured at 517 nm using a UV-visible spectrophotometer. The percentage inhibition of the DPPH radical (scavenging activity) was calculated according to the following formula:

(3) $$\begin{array}{c} \text{Scavenging}\;\text{effect}\;\;\left(\%\right)=\frac{A_{c}-A_{s}}{A_{c}}\times 100, \end{array}$$

where *A*_*c*_ is the absorbance of the control reaction and *A*_*s*_ is the absorbance of the sample extract. Sample concentration providing 50% inhibition (IC_50_) was calculated from the graph plotting inhibition percentage against protease concentration. A lower absorbance of the reaction mixture indicated a higher DPPH radical-scavenging activity. Butylated hydroxyanisole (BHA) was used as a standard. The test was carried out in triplicate.

#### 2.16.2. Assay of Superoxide Radical-(O_2_^−^)-Scavenging Activity

The assay was based on the capacity of the antioxidant to inhibit formazan formation by scavenging the superoxide radicals generated in riboflavin-light-NBT system [22]. The method used by Martinez et al. [23] for determination of superoxide dismutase was followed after modifications [23]. Each 3 mL of reaction mixture contained 50 mM sodium phosphate buffer, pH 8.0, 13 mM methionine, 2 *μ*M riboflavin, 100 *μ*M EDTA, NBT (75 *μ*M), and 1 mL of the protease of different concentrations. The production of blue formazan was followed by monitoring the increase in absorbance at 560 nm after a 10 min illumination from a fluorescent lamp. The entire reaction assembly was enclosed in a box lined with aluminium foil. Identical tubes with reaction mixture were kept in the dark and served as blanks. The percentage inhibition of superoxide anion generation was calculated using the following formula:

(4) $$\begin{array}{c} \left(\%\right)\;\;\text{Inhibition}=\frac{A_{c}-A_{s}}{A_{c}}\times 100, \end{array}$$

where *A*_*c*_ is the absorbance of the control and *A*_*s*_ is the absorbance of the protease.

#### 2.16.3. Assay of Nitric Oxide-Scavenging Activity

The procedure is based on the principle that sodium nitroprusside in aqueous solution at physiological pH spontaneously generates nitric oxide which interacts with oxygen to produce nitrite ions that can be estimated using the Griess reagent. Scavengers of nitric oxide compete with oxygen, leading to reduced production of nitrite ions. For the experiment, sodium nitroprusside (10 mM), in phosphate-buffered saline, was mixed with different concentrations of protease dissolved in water and incubated at room temperature for 150 min. The same reaction mixture, without the protease but with an equivalent amount of water, served as a control. After the incubation period, 0.5 mL of the Griess reagent (1% sulfanilamide, 2% H_3_PO_4_, and 0.1% *N*-(1-naphthyl) ethylenediamine dihydrochloride) was added. The absorbance of the chromophore formed was read at 546 nm. Ascorbic acid was used as a positive control [24].

#### 2.16.4. Reducing Power Determination

The reducing power of protease was determined according to the method [25]. Different amounts of protease in water were mixed with phosphate buffer (2.5 mL, 0.2 M, pH 8.0) and potassium ferricyanide (K_3_Fe(CN)_6_) (2.5 mL, 1%). The mixture was incubated at 50°C for 20 min. A portion (2.5 mL) of trichloroacetic acid (10%) was added to the mixture, which was then centrifuged for 10 min. The upper layer of solution (2.5 mL) was mixed with distilled water (2.5 mL) and FeCl_3_ (0.5 mL, 0.1%), and the absorbance was measured at 700 nm. Increased absorbance of the reaction mixture indicated increased reducing power.

#### 2.16.5. Metal Chelating Activity

Chelating property of ferrous ions by the protease was estimated using the standard method [26]. Briefly, the protease was added to a solution of 2 mM FeCl_2_ (0.05 mL). The reaction was initiated by the addition of 5 mM ferrozine (0.2 mL); the mixture was shaken vigorously and left standing at room temperature for 10 min. Absorbance of the solution was then measured spectrophotometrically at 562 nm. Percentage inhibition of ferrozine-Fe^2+^ complex formation was calculated as ((*A*_*c*_ − *A*_*s*_)/*A*_*c*_) × 100, where *A*_*c*_ was the absorbance of the control and   *A*_*s*_ was the absorbance of the protease/standard.

### 2.17. Data Handling

Results have been expressed as means ± standard deviations of four replicated determinations. Minitab software (Minitab package version 16.0, Inc., USA) was used for data analysis.

## 3. Results and Discussion

### 3.1. Isolation, Identification, and Enzyme Production of a Marine Actinobacterium

**Figure 1:**
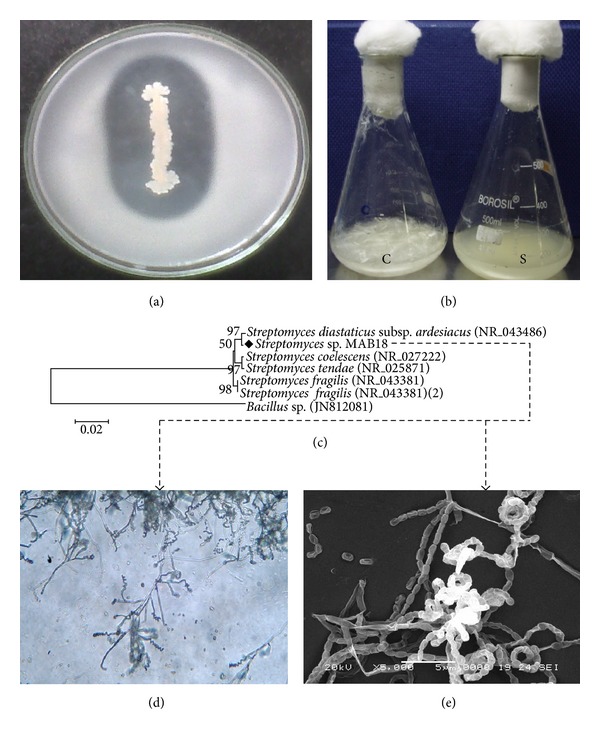
(a) Screening of protease enzyme on gelatin agar medium. (b) Feather degradation by *Streptomyces* sp. MAB18 in liquid media with the whole intact feather as sole carbon and nitrogen source. C: uninoculated medium (control), clear with intact feathers. S: complete hydrolysis of feathers inoculated with MAB18. (c) Phylogenetic tree of the 16S rDNA sequence of strain MAB18 and related strains. (d) Micromorphology of spore chains of the *Streptomyces* sp. MAB18 (×400). (e) Scanning electron micrograph of spores of the *Streptomyces* sp. MAB18 (×5000).

A marine actinobacterial strain MAB18 strain was isolated from the marine sediments of Cuddalore coast, India. It formed a clear zone on gelatin agar plates around the colony. Figure 1(a) indicates that the MAB18 strain has produced the most pronounced clearing zone. This isolate was pursued for identification, protease enzyme production, and the ability to degrade feathers. The degradation of whole-chicken feather by *Streptomyces* sp. MAB18 is shown in Figure 1(b). Degradation was observed after incubation at 50°C for 5 days. Mohamedin [7] reported that* Streptomyces *degraded whole intact chicken feather at 50°C [7]. 

The strain MAB18 showed the presence of LL-diaminopimelic acid and glycine in the cell wall and there was no characteristic sugar pattern and hence the strain belonged to the cell wall type I. Its aerial mycelium was grey. Sporophores were spiral; hooks and loops were also formed (Figure 1(d)). Conidia were oblong, the surface of which was smooth (Figure 1(e)). This strain was identified as *Streptomyces* sp. MAB18, based on its morphological, physiological, and biochemical characteristics and it was confirmed by the 16S rDNA sequencing (Figure 1(c)). The sequence was submitted to Gene Bank in NCBI (http://www.ncbi.nlm.nih.gov/nuccore/JQ068140.1) with the accession number JQ068140. 

### 3.2. Optimization of Protease Production

#### 3.2.1. Selection of Physicochemical Parameters, Carbon, and Nitrogen Sources by One-Variable-at-a-Time Approach

Effect of carbon and nitrogen sources on enzyme yield is shown in Table S3A.   *Streptomyces* sp. MAB18 produced higher amount of protease in the presence of glucose, maltose, and sucrose. However, production of protease was higher (165 U/mL) in the medium which contained glucose than that with maltose (145.03 U/mL) and sucrose (123.57 U/mL). Nitrogen sources including casein, yeast extract, soybean meal, and peptone significantly influenced the enzyme production. Among these, casein proved to be a good nitrogen source for stimulating protease production (156.80 U/mL). Among the various carbon and nitrogen sources tested, glucose and casein were found to be the most suitable substrates for protease production. Thus, these substrates were selected for further optimization steps. The highest protease production was observed in glucose and casein, which is in conformity with an earlier study [12].

#### 3.2.2. Screening of Parameters Using the Plackett-Burman Design

Experiment was conducted in 12 runs to study the effect of the selected variables. Table S1A represents the results of the screening experiments using the Plackett-Burman design. Statistical analysis of the responses was performed which is represented in Table S1B. The model *F* value of 3790.63 implies that the model is significant. Values of *Prob *< 0.05 indicate that the model terms are significant.

Magnitude of the effects indicates the level of the significance of the variables on protease production. Among the variables of screened whole-chicken feather (WCF), peptone, beef extract, K_2_HPO_4_, MgSO_4_·7H_2_O, CaCl_2_, Na_2_CO_3_, and NaCl were identified as the most significant variables, influencing protease production (Table S1C).

#### 3.2.3. Optimization of Significant Variables Using Response Surface Methodology (RSM)

 Experiments conducted in the present study were targeted towards the construction of a quadratic model consisting of twenty-seven trials. The design matrix and the corresponding results of RSM experiments to determine the effects of four independent variables (whole-chicken feather (WCF), peptone, NaCl, and Na_2_CO_3_) are shown in Table S2A, along with the mean predicted values. The regression analysis of the optimization study indicated that the model terms, *X*_1_, *X*_2_, *X*_3_, *X*_4_, *X*_1_^2^, *X*_2_^2^, *X*_3_^2^, *X*_4_^2^, *X*_1_ *X*_2_, *X*_1_ *X*_3_, *X*_1_ *X*_4_, *X*_2_ *X*_3_, *X*_2_ *X*_4_ and *X*_3_ *X*_4_, were significant (*P* < 0.05). These results indicate that the concentration of the whole-chicken feather (WCF), peptone, NaCl, and Na_2_CO_3_ bears a direct relationship with protease production. The interactions between whole-chicken feather (WCF), peptone, NaCl and Na_2_CO_3_ were significant, as shown by the low *P* values *P* < 0.0001 and *P* < 0.0002, respectively. Analysis of variance (ANOVA) (Table S2B) depicts the *P* values for the model (*P* < 0.0001) and for lack of fit also suggested that the obtained experimental data are a good fit with the model.

Regression equation coefficients were calculated and the data were fitted to a second-order polynomial equation. The response, protease (*Y*) by *Streptomyces* sp. MAB18, can be expressed in terms of the following regression equation:

(5) Y=25.6972+3.7770X1+10.2709X2  +10.8862X3+11.3939X4−0.3154X12−1.6146X22−1.6599X32−1.5509X42+0.1756X1X2+0.0527X1X3−0.1130X1X4−0.0051X2X3−0.0940X2X4+0.0233X3X4,

where whole chicken feather (WCF) (*X*_1_), peptone (*X*_2_), NaCl (*X*_3_), Na_2_CO_3_ (*X*_4_), whole chicken feather∗whole chicken feather (*X*_1_^2^), peptone∗peptone (*X*_2_^2^), NaCl∗NaCl (*X*_3_^2^), Na_2_CO_3_∗Na_2_CO_3_ (*X*_4_^2^), whole chicken feather*peptone (*X*_1_ *X*_2_), whole chicken feather∗NaCl (*X*_1_ *X*_3_), whole chicken feather∗Na_2_CO_3_ (*X*_1_ *X*_4_), peptone∗NaCl (*X*_2_ *X*_3_), peptone∗Na_2_CO_3_ (*X*_2_ *X*_4_), and NaCl∗Na_2_CO_3_ (*X*_3_ *X*_4_).

 Regression equation obtained from the ANOVA showed that the *R* ^2^ (multiple correlation coefficient) was 0.999 5 (a value >0.80 indicates fitness of the model). This is an estimate of the fraction of overall variation in the data accounted by the model, and thus the model is capable of explaining 99.95% of the variation in response. The “adjusted *R* ^2^” was 0.998 8 and the “predicted *R* ^2^” was 0.996 8, and this indicates that the model is good.

 In order to determine the optimal levels of each variable for maximum protease production, three-dimensional response surface plots were constructed by plotting the response (protease production) on the *z*-axis against any two independent variables, while maintaining other variables at their central levels (Figure  S1). Maximum protease production was obtained at the middle level of each pair of factors at a constant middle level of the other factor. Further increase in these factors above the middle level showed a decrease in protease production. In order to determine the maximum protease production corresponding to the optimum levels of whole-chicken feather, peptone, NaCl, and Na_2_CO_3_, a second-order polynomial model was used to calculate the values of these variables. Fitting of the experimental data to equation allowed determination of the levels of whole-chicken feather (*X*_1_ = 6.565 6 g), peptone (*X*_2_ = 3.364 3 g), NaCl (*X*_3_ = 3.400 7 g), and Na_2_CO_3_ (*X*_4_ = 3.437 2 g), giving a maximum protease concentration of 92.37 U/mL in shake flask culture.

Fermentation was performed using 100 mL synthetic medium containing the optimized level of whole chicken feather (6.565 6 g), peptone (3.364 3 g), NaCl (3.400 7 g) and Na_2_CO_3_ (3.437 2). Maximum protease production (94.84 U/mL) was obtained, which was slightly higher than the value given by the model.

### 3.3. Time Course of Protease Production

**Figure 2:**
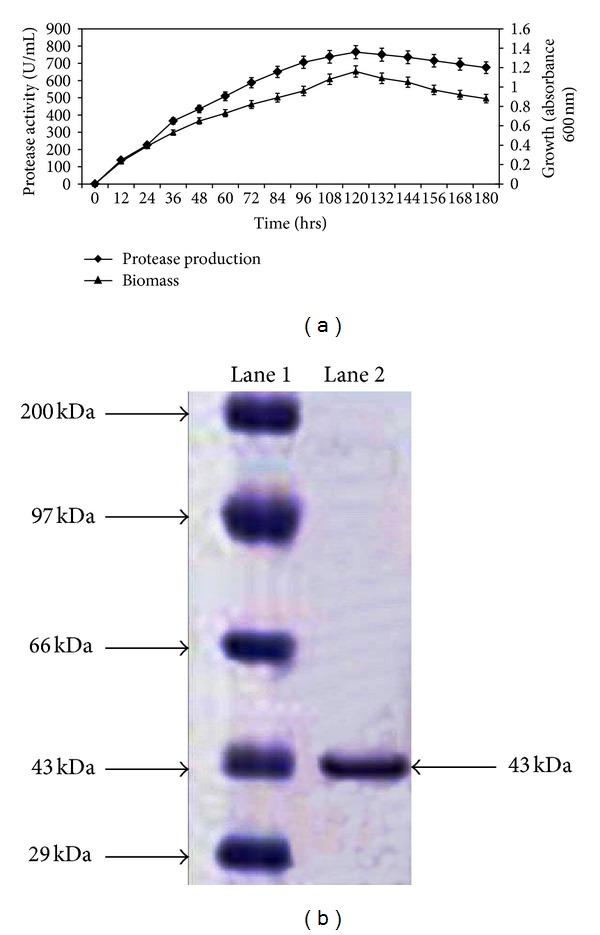
(a) Time course of protease production from *Streptomyces* sp. MAB18. (b) SDS-PAGE analysis of protease from *Streptomyces* sp. MAB18. Lane 1, molecular markers (29–200 kDa); lane 2, purified enzyme.

The time course was studied up to 180 hrs in optimized medium and maximum enzyme production of 765 U/mL was achieved in 120 hrs of incubation (Figure 2(a)). It was observed that the enzyme production was built up slowly during the exponential phase and it attained the maximum at the onset of the stationary phase, thus lending support to earlier study [27]. 

### 3.4. Purification of Protease

Protease from the culture broth of *Streptomyces* sp. MAB18 was purified through multistep purification and summary of the purification profile is presented in Table S3B. The overall purification fold of protease was about 17.13 with the specific activity of 2398.36 U/mg and 21.62% yield. Homogeneity of the purified enzyme was analyzed and confirmed by the single band obtained in SDS-PAGE. Molecular weight of the purified protease was estimated as 43 kDa (Figure 2(b)) and it is worth mentioning here that different molecular masses of protease ranging from 23 to 24 kDa have been reported for *Nesterenkonia *sp. AL-20 and *Bacillus pseudofirmus *Al-89 [12].

### 3.5. Effect of Temperature, pH, and NaCl on Purified Enzyme

**Figure 3:**
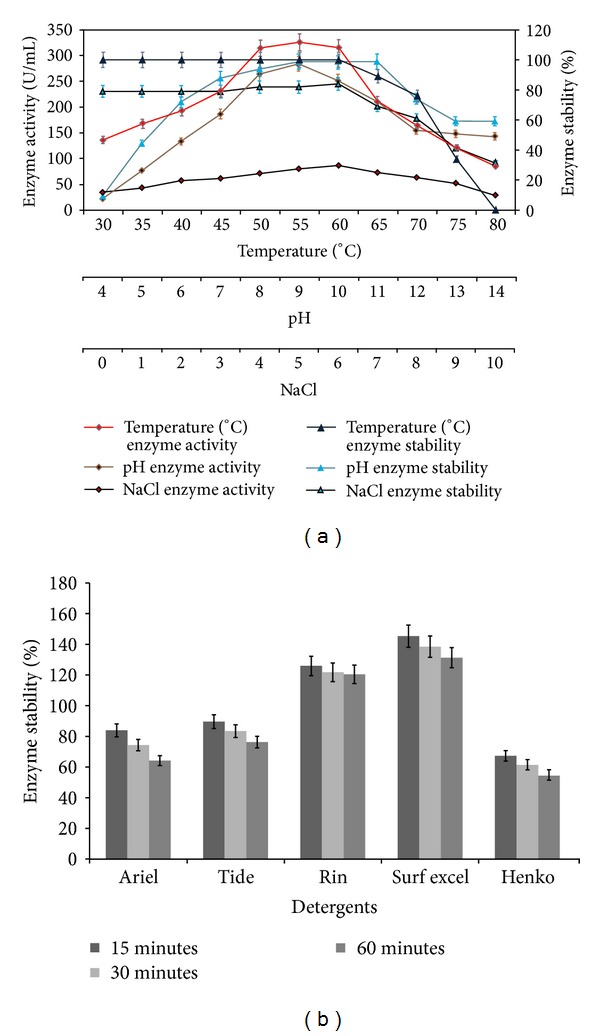
(a) Effect of temperature, pH and sodium chloride concentrations on enzyme activity and stability of purified protease from *Streptomyces* sp. MAB18. The values are mean ± SD, *n* = 3. Absence of bars indicates that errors were smaller than symbols. (b) Detergents stability and compatibility of the protease *Streptomyces* sp. MAB18 in commercial detergents. The values are mean ± SD, *n* = 3.

Optimum temperature and pH for the protease activity of *Streptomyces* sp. MAB18 were 50–60°C and 8–10, respectively (Figure 3(a)). Thermostability study showed that the enzyme was 100% stable up to 60°C for 1 h, 89% at 65°C for 1 h and 76% at 70°C for 1 h, and it appears that the enzyme of *Streptomyces* sp. MAB18 is considerably more stable than the other *Streptomyces* sp. protease reported to date [7, 28], as most of the other enzymes were stable only up to 30–60°C. Likewise, the enzyme from *Streptomyces* sp. MAB18 was stable up to pH 12.0; it retained 100% activity at pH 9–11 and 75% activity even after 1 h incubation at 70°C and pH 12.0 (Figure 3(a)). Previously, researchers have reported that protease was stable only between pH 4 and 11.0 [7, 28].

Protease from *Streptomyces* sp. MAB18 showed activity over a broad range of NaCl concentration (0–10%) and the optimal concentration was 6% (Figure 3(a)). The enzyme retained a good 89% activity in 6% NaCl concentration, but the enzyme activity decreased when the NaCl concentration increased from 8 to 10%. At 10% NaCl concentration, the enzyme retained 30% of its original activity after 12 h incubation (Figure 3(a)). Even recent reports reveal that the protease activity with an optimal 5% NaCl tolerance was shown by *Streptomyces clavuligerus *strain Mit-1 and *Nocardiopsis kunsanensis *sp. [29, 30]. Thus, the high salt tolerance shown by the MAB18 enzyme will have a number of applications in the biotechnological processes that depend on higher salinity or osmotic pressure for long periods of incubation including the potential applications in treating the agricultural wastes, bioremediation of keratin materials for sustainable bio-based production, and bioenergy production.

### 3.6. Effect of Metal Ions and Chemicals on Enzyme Activity

Effect of various metal ions on the protease activity of the purified protease enzyme is shown in Table S4. Activity of the protease was strongly inhibited by Hg^2+^ and Pb^2+^ in 1–10 mM concentrations. When treated with Fe^2+^ and Mg^2+^, the enzyme activity was stimulated, as reported earlier [31]. Ag^2+^, Ca^2+^, and Mn^2+^ slightly inhibited the enzyme activity. The stimulatory effect of Fe^2+^ and Mg^2+^ at 1–10 mM concentrations on protease suggests that these metals can act as cofactors in increasing the enzyme activity.

Presence of Triton X-100, DMSO, sodium sulphite, and *β*-mercaptoethanol also stimulated the protease activity. SDS, 1,10-phenanthroline, and EDTA strongly inhibited the enzyme activity and Tween-80 and isopropanol slightly inhibited the activity (Table S4). Jain et al. [19] also reported that *β*-mercaptoethanol enhanced the enzyme activity and EDTA strongly inhibited the activity in *Bacillus *sp. [19]. 

### 3.7. Compatibility of Protease with Commercial Laundry Detergents

*Streptomyces* sp. MAB18 protease showed good stability with the commercial detergents tested (Figure 3(b)). It retained 64.2% residual activity after 1 h of incubation in Ariel, 76.2% in Tide, 120.4% in Rin, 131.3% in Surf excel and 54.4% in Henko. After 15 min, it retained 83.9%, 89.6%, 125.9%, 145.3% and 67.3%, respectively. The maximum stability was observed with Surf excel and Rin. Similar trend has been reported by Mukherjee et al. [20].

### 3.8. Antioxidant Activity of Protease

Protease was assayed for its antioxidant activity using DPPH radical-scavenging activity, O_2_-scavenging activity, NO-scavenging activity, Fe^2+^ chelating activity, and reducing power.

#### 3.8.1. DPPH Free Radical-Scavenging Activity

**Figure 4:**
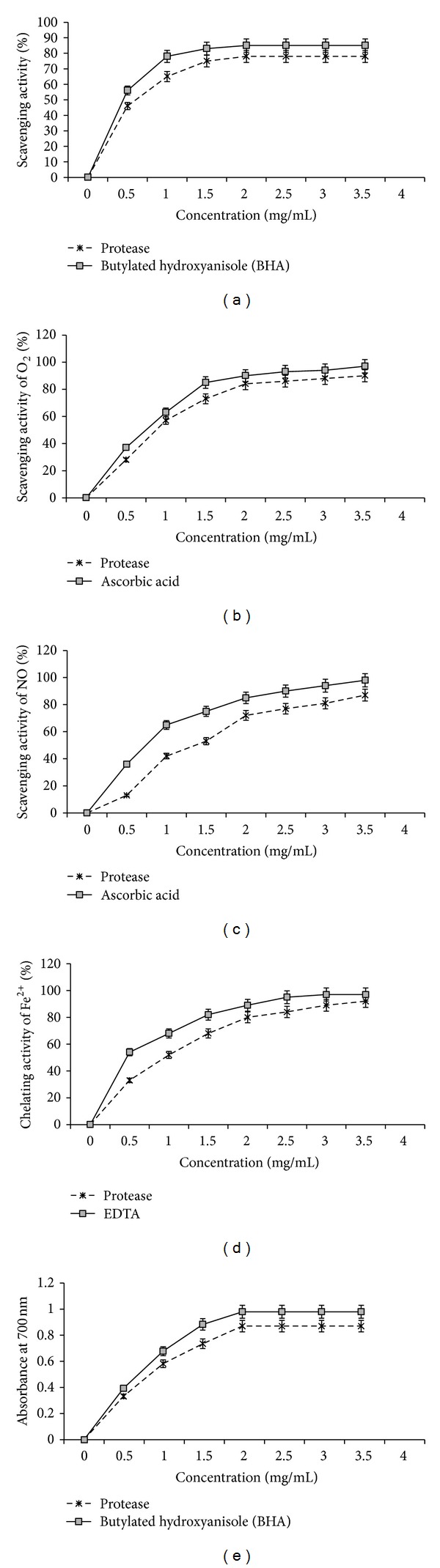
Antioxidant activity of protease, (a) DPPH-scavenging activity; (b) O_2_-scavenging activity; (c) NO-scavenging activity; (d) Fe^2+^ chelating activity; and (e) reducing power.

DPPH is a stable free radical that shows maximum absorbance at 517 nm. When DPPH radicals encounter a proton-donating substrate such as an antioxidant, the radicals would be scavenged and the absorbance would be reduced [32]. The decrease in absorbance is taken as a measure for radical-scavenging activity. The DPPH radical-scavenging activity was investigated at different concentrations (0–3.5 mg/mL) of the protease. The results presented in Figure 4(a) clearly show that the protease exhibited an interesting radicals scavenging activity with an IC_50_ value of 78 ± 0.28 mg/mL.

#### 3.8.2. Assay of Superoxide Radical (O_2_^−^)-Scavenging Activity

Figure 4(b) shows the superoxide radical-(O_2_^−^)-scavenging activity of the protease, as measured by the riboflavin-NBT-light system *in vitro*. Superoxide radical is known to be very harmful to cellular components as a precursor of more reactive oxygen species [33]. Photochemical reduction of flavins generates O_2_^−^, which reduces NBT, resulting in the formation of blue formazan [22]. The protease was found to be a moderate scavenger of superoxide radical generated in riboflavin-NBT-light system *in vitro*. The protease inhibited the formation of the blue formazan and the % inhibition was proportional to the concentration with an IC_50_ value of 84 mg/mL. These results indicated that the tested protease had a notable effect on scavenging of superoxide when compared with ascorbic acid, which was used as a positive control.

#### 3.8.3. Assay of Nitric Oxide-Scavenging Activity

 Protease also showed a moderate nitric oxide-scavenging activity at different concentrations, 0.5–3.5 mg/mL, in a dose dependent manner (IC_50_ = 72 mg/mL) (Figure 4(c)). Nitric oxide is an essential bioregulatory molecule required for several physiological processes like regulation of blood pressure, prevention of aggregation and adhesion of platelets, assisting the immune system to kill a wide variety of pathogens and block viral replication, promotion of certain types of cancer, promotion of penile erection and spermatogenesis, and facilitating childbirth [34]. In addition to reactive oxygen species, nitric oxide is also implicated in inflammation, cancer and other pathological conditions [35]. Present results suggest that *Streptomyces* sp. MAB18 is a potent and novel source of therapeutic agents for scavenging NO and regulating the pathological conditions caused by excessive generation of NO as its protease showed a moderate nitric oxide-scavenging activity. Percent inhibition was increased with the increasing concentration of the protease. 

#### 3.8.4. Reducing Power Determination

Figure 4(e) shows the reductive capabilities of protease compared to BHA. For the measurements of the reductive ability, we investigated the Fe^3+^-Fe^2+^ transformation in the presence of protease, using the standard method [25]. Earlier authors [36, 37] have observed a direct correlation between antioxidant activities and reducing power of certain protease. The reducing properties are generally associated with the presence of reductones [38], which have been shown to exert antioxidant action by breaking the free radical chain by donating a hydrogen atom. Reductones are also reported to react with certain precursors of peroxide, thus preventing peroxide formation. Our data on the reducing power of protease suggest that it is likely to contribute significantly towards the observed antioxidant effect. However, the antioxidant activity of compounds has been attributed to various mechanisms, such as prevention of chain initiation, binding of transition metal ion catalysts, decomposition of peroxides, and prevention of continued hydrogen abstraction [39]. Similar to the antioxidant activity, reducing power of protease increased with the increasing amount of sample. However, the reducing power of BHA was relatively more pronounced than that of protease. 

#### 3.8.5. Metal Chelating Activity

Chelation of ferrous ions by protease was estimated by the standard method [26]. Ferrozine can quantitatively form complexes with Fe^2+^. In the presence of other chelating agents, the complex formation is disrupted with the result that the red colour of the complexes decreases. Measurement of the rate of colour reduction therefore allows estimation of the chelating activity of the coexisting chelator [40]. In the present assay, both protease and EDTA interfered with the formation of ferrous and ferrozine complex, suggesting that it has chelating activity and captures ferrous ion before ferrozine. Absorbance of Fe^2+^-ferrozine complex was decreased dose dependently; otherwise, the activity was increased on increasing concentration from 0 to 3.5 mg/mL. Metal chelating capacity was significant since the protease reduced the concentration of the catalyzing transition metal in lipid peroxidation [41]. It was reported that chelating agents, which form *σ*-bonds with a metal, are effective as secondary antioxidants because they reduce the redox potential, thereby stabilising the oxidized form of the metal ion. The data obtained and shown in Figure 4(d) reveal that the protease has an effective capacity for iron binding, suggesting that its action as an antioxidant may be related to its iron binding capacity. 

## 4. Conclusion

In the present study, feather degradation was successfully carried out by fermentation of the strain *Streptomyces* sp. MAB18. Production of protease from this strain was simple and it will be easy to scale up, as this actinobacterium grows on simple media with feathers as a sole source of carbon, nitrogen, and energy, thus allowing its enzyme production from an inexpensive substrate and a commercial potential with low production cost. When the protease obtained from MAB18 under optimum conditions was assessed for antioxidant activity using DPPH radical-scavenging activity, O_2_-scavenging activity, NO-scavenging activity, Fe^2+^ chelating activity, and reducing power, the enzyme was found to possess good antioxidant potential. Hence, the use of this protease in fish feed formulations as a source of protein and natural antioxidants would be an advantage both for the aquaculture industry and the consumers. Further research is essential to incorporate this protease in animal models to study its effect on the growth and *in vivo* lipid peroxidation. Further, the oxidative stability of the animal diet formulated using the protease should also be given due attention.

## Supplementary Material

Figure S1: Optimization of Significant Variables Using Response Surface Methodology (RSM): The statistical optimization of protease production using RSM. (A) peptone and Na_2_CO_3_; (B) NaCl and Na_2_CO_3_; (C) WCF and peptone; (D) peptone and NaCl; (E) WCF and Na_2_CO_3_; (F) WCF and NaCl.
Table S1: Screening of Parameters Using the Plackett-Burman Design: Plackett-Burman experimental design matrix with protease production levels. Statistical analysis of the model. Statistical parameters for selected the linear polynomial model using Plackett-Burman design.
Table S2: Optimization of Significant Variables Using Response Surface Methodology (RSM): Central composite factor experimental design along with experimental and predicted values. Analysis for variance of protease production.
Table S3 A: Optimization of Protease Production: Effect of various carbon and nitrogen sources on production of protease from *Streptomyces* sp. MAB18.
Table S3 B: Purification of Protease: Summary of purification steps of protease from *Streptomyces* sp. MAB18.
Table S4: Effect of Metal Ions and Chemicals on Enzyme Activity: Effect of metal ions and chemicals on activity of the purified protease from *Streptomyces* sp. MAB18.
