# Supplementary material for: Production, Characterization and Antioxidant Potential of Protease from Streptomyces sp. MAB18 Using Poultry Wastes
Source: Biomed Res Int. 2013 Aug 7;2013:496586. doi: 10.1155/2013/496586 (PMC3749541; doi:10.1155/2013/496586)
Supplement: Supplementary file 1 — Figure S1: Optimization of Significant Variables Using Response Surface Methodology (RSM): The statistical optimization of protease production using RSM. (A) peptone and Na2CO3; (B) NaCl and Na2CO3; (C) WCF and peptone; (D) peptone and NaCl; (E) WCF and Na2CO3; (F) WCF and NaCl. Table S1: Screening of Parameters Using the Plackett-Burman Design: Plackett-Burman experimental design matrix with protease production levels. Statistical analysis of the model. Statistical parameters for selected the linear polynomial model using Plackett-Burman design. Table S2: Optimization of Significant Variables Using Response Surface Methodology (RSM): Central composite factor experimental design along with experimental and predicted values. Analysis for variance of protease production. Table S3 A: Optimization of Protease Production: Effect of various carbon and nitrogen sources on production of protease from Streptomyces sp. MAB18. Table S3 B: Purification of Protease: Summary of purification steps of protease from Streptomyces sp. MAB18. Table S4: Effect of Metal Ions and Chemicals on Enzyme Activity: Effect of metal ions and chemicals on activity of the purified protease from Streptomyces sp. MAB18. [file 496586.f1.docx]

**Supplementary materials**

**
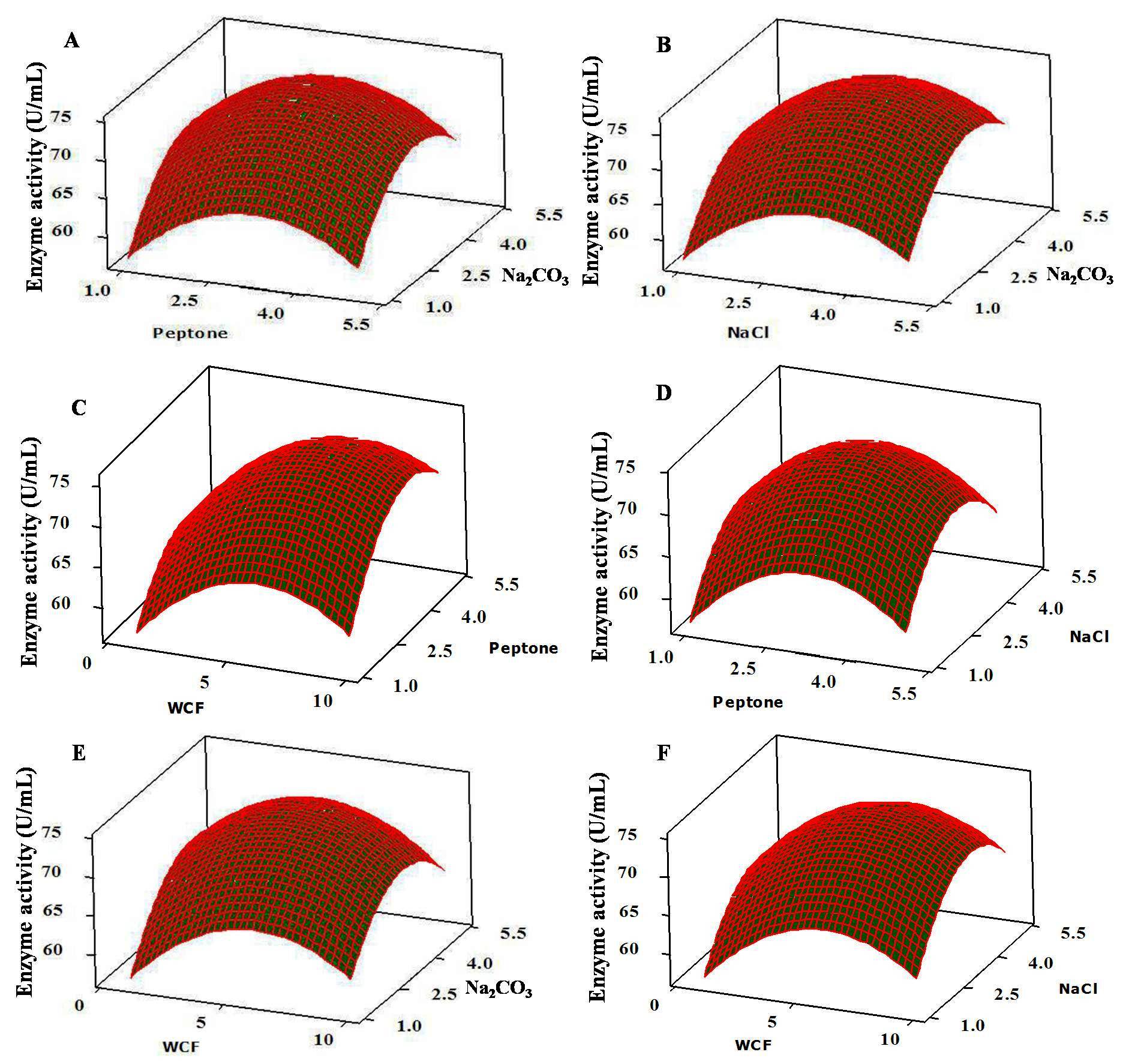
**

**Fig. S1. Statistical optimization of protease production using RSM. (A) peptone and Na_2_CO_3_; (B) NaCl and Na_2_CO_3_; (C) WCF and peptone; (D) peptone and NaCl; (E) WCF and Na_2_CO_3_; (F) WCF and NaCl.**


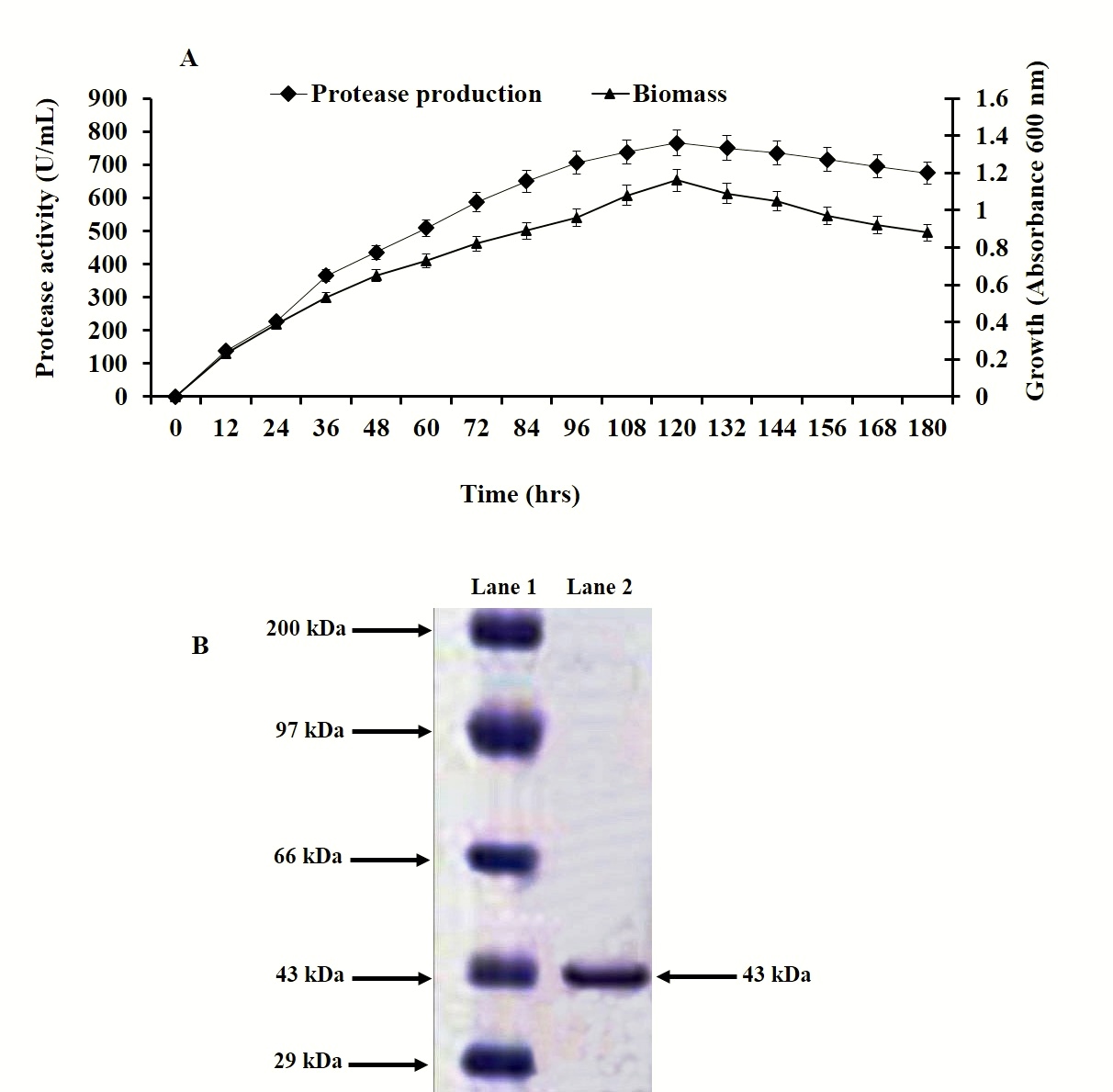


**Fig. S2. (A) Time course of protease production from *Streptomyces* sp. MAB18. (B) SDS-PAGE analysis of protease from *Streptomyces* sp. MAB18. Lane 1, Molecular markers (29-200 kDa); lane 2, Purified enzyme.**

**Table S1 A. Plackett–Burman experimental design matrix with protease production levels.**

| **Std** | **WCF** | **Peptone** | **Beef**  **extract** | | **K_2_HPO_4_** | | **MgSO_4_.7H_2_O** | | **CaCl_2_** | **Na_2_CO_3_** | | | **NaCl** | **Protease Activity (U/mL)** | | | |
| --- | --- | --- | --- | --- | --- | --- | --- | --- | --- | --- | --- | --- | --- | --- | --- | --- | --- |
|  |  |  |  |  |  |  |  |  |  |  |  |  |  | **Observed^a^** | | **Predicted** | |
| 1 | 10 | 1 | 5 | | 0.5 | | 0.1 | | 0.1 | 5 | | | 5 | 58.560 | | 58.4605 | |
| 2 | 10 | 5 | 1 | | 1.0 | | 0.1 | | 0.1 | 1 | | | 5 | 64.360 | | 64.3085 | |
| 3 | 1 | 5 | 5 | | 0.5 | | 0.5 | | 0.1 | 1 | | | 1 | 40.820 | | 40.9762 | |
| 4 | 10 | 1 | 5 | | 1.0 | | 0.1 | | 0.5 | 1 | | | 1 | 61.190 | | 61.4972 | |
| 5 | 10 | 5 | 1 | | 1.0 | | 0.5 | | 0.1 | 5 | | | 1 | 85.354 | | 85.4055 | |
| 6 | 10 | 5 | 5 | | 0.5 | | 0.5 | | 0.5 | 1 | | | 5 | 72.210 | | 72.0538 | |
| 7 | 1 | 5 | 5 | | 1.0 | | 0.1 | | 0.5 | 5 | | | 1 | 91.320 | | 91.0128 | |
| 8 | 1 | 1 | 5 | | 1.0 | | 0.5 | | 0.1 | 5 | | | 5 | 68.370 | | 68.4695 | |
| 9 | 1 | 1 | 1 | | 1.0 | | 0.5 | | 0.5 | 1 | | | 5 | 76.350 | | 76.2505 | |
| 10 | 10 | 1 | 1 | | 0.5 | | 0.5 | | 0.5 | 5 | | | 1 | 87.390 | | 87.3385 | |
| 11 | 1 | 5 | 1 | | 0.5 | | 0.1 | | 0.5 | 5 | | | 5 | 95.450 | | 95.7572 | |
| 12 | 1 | 1 | 1 | | 0.5 | | 0.1 | | 0.1 | 1 | | | 1 | 35.320 | | 35.1638 | |
| **B. Statistical analysis of the model** | | | | | | | | | | | | | | | | | |
| **Sources** | | | **DF** | | **Seq SS** | | **Adj SS** | | **Adj MS** | | | | ***F*** | | | ***P*** | |
| Main Effects | | | 8 | | 3981.44 | | 3981.44 | | 497.68 | | | | 3790.63 | | | 0.000 | |
| Residual Error | | | 3 | | 0.39 | | 0.39 | | 0.13 | | | | - | | | - | |
| Total | | | 11 | | 3981.83 | | - | | - | | | | - | | | - | |
| **C. Statistical parameters for selected the linear polynomial model using Plackett-Burman design** | | | | | | | | | | | | | | | | | |
| **Term** | | | | | **Effect** | | **Coef** | | **SE Coef** | | | ***t-*value** | | | | ***P*** | |
| Constant | | | | | - | | 69.713 | | 0.1046 | | | 666.59 | | | | 0.000^b^ | |
| WCF | | | | | 3.572 | | 1.786 | | 0.1046 | | | 17.08 | | | | 0.000^b^ | |
| Peptone | | | | | 10.389 | | 5.194 | | 0.1046 | | | 49.66 | | | | 0.000^b^ | |
| Beef extract | | | | | -8.626 | | -4.313 | | 0.1046 | | | -41.23 | | | | 0.000^c^ | |
| K_2_HPO_4_ | | | | | 9.532 | | 4.313 | | 0.1046 | | | 45.57 | | | | 0.000^b^ | |
| MgSO_4_.7H_2_O | | | | | 4.049 | | 2.024 | | 0.1046 | | | 19.35 | | | | 0.000^b^ | |
| CaCl_2_ | | | | | 21.854 | | 10.927 | | 0.1046 | | | 104.47 | | | | 0.000^b^ | |
| Na_2_CO_3_ | | | | | 22.699 | | 11.350 | | 0.1046 | | | 108.50 | | | | 0.000^b^ | |
| NaCl | | | | | 5.651 | | 2.826 | | 0.1046 | | | 27.01 | | | | 0.000^b^ | |

*R^2^=* 99.99; Adj-*R^2^ =*99.96*;* Pred-*R^2^ =* 99.84*;*

Non-significant at *P*<0.05.

*^a^ The observed values of protease production were the mean values of duplicates*

^b^ Significant positive effect.

^c^ Significant negative effect.

**Table S2 A. Central composite factor experimental design along with experimental and predicted values**

| **Experimental number** | | **WCF** | **Peptone** | | **NaCl** | | **Na_2_CO_3_** | | **Protease activity (U/mL)** | | | |
| --- | --- | --- | --- | --- | --- | --- | --- | --- | --- | --- | --- | --- |
|  |  |  |  |  |  |  |  |  | **Observed^a^** | | **Predicted** | |
| 1 | | 1.0 | 1 | | 3 | | 3 | | 75.786 | | 75.6619 | |
| 2 | | 10.0 | 1 | | 3 | | 3 | | 78.362 | | 78.3831 | |
| 3 | | 1.0 | 5 | | 3 | | 3 | | 77.372 | | 77.5054 | |
| 4 | | 10.0 | 5 | | 3 | | 3 | | 86.271 | | 86.5496 | |
| 5 | | 5.5 | 3 | | 1 | | 1 | | 74.263 | | 74.5718 | |
| 6 | | 5.5 | 3 | | 5 | | 1 | | 79.362 | | 79.4691 | |
| 7 | | 5.5 | 3 | | 1 | | 5 | | 79.352 | | 79.3994 | |
| 8 | | 5.5 | 3 | | 5 | | 5 | | 84.821 | | 84.6668 | |
| 9 | | 1.0 | 3 | | 3 | | 1 | | 73.382 | | 73.3143 | |
| 10 | | 10.0 | 3 | | 3 | | 1 | | 81.372 | | 81.2325 | |
| 11 | | 1.0 | 3 | | 3 | | 5 | | 80.362 | | 80.3625 | |
| 12 | | 10.0 | 3 | | 3 | | 5 | | 84.281 | | 84.2097 | |
| 13 | | 5.5 | 1 | | 1 | | 3 | | 74.321 | | 74.2078 | |
| 14 | | 5.5 | 5 | | 1 | | 3 | | 79.352 | | 79.2538 | |
| 15 | | 5.5 | 1 | | 5 | | 3 | | 79.372 | | 79.3312 | |
| 16 | | 5.5 | 5 | | 5 | | 3 | | 84.321 | | 84.2952 | |
| 17 | | 1.0 | 3 | | 1 | | 3 | | 74.372 | | 74.3363 | |
| 18 | | 10.0 | 3 | | 1 | | 3 | | 79.378 | | 79.2689 | |
| 19 | | 1.0 | 3 | | 5 | | 3 | | 78.375 | | 78.4686 | |
| 20 | | 10.0 | 3 | | 5 | | 3 | | 85.281 | | 85.3013 | |
| 21 | | 5.5 | 1 | | 3 | | 1 | | 74.291 | | 74.3229 | |
| 22 | | 5.5 | 5 | | 3 | | 1 | | 80.321 | | 80.0804 | |
| 23 | | 5.5 | 1 | | 3 | | 5 | | 79.863 | | 80.0881 | |
| 24 | | 5.5 | 5 | | 3 | | 5 | | 84.388 | | 84.3406 | |
| 25 | | 5.5 | 3 | | 3 | | 3 | | 92.371 | | 92.3707 | |
| 26 | | 5.5 | 3 | | 3 | | 3 | | 92.370 | | 92.3707 | |
| 27 | | 5.5 | 3 | | 3 | | 3 | | 92.371 | | 92.3707 | |
| **B. Analysis for variance of protease production** | | | | | | | | | | | | |
| **Source** | **DF** | | | **Seq SS** | | **Adj SS** | | **Adj MS** | | ***F*** | | ***P*** |
| Regression | 14 | | | 788.232 | | 788.232 | | 56.302 | | 1563.42 | | 0.000 |
| Linear | 4 | | | 331.838 | | 331.838 | | 82.960 | | 2303.65 | | 0.000 |
| Square | 4 | | | 440.751 | | 440.751 | | 110.817 | | 3059.73 | | 0.000 |
| Interaction | 6 | | | 15.643 | | 15.643 | | 2.607 | | 72.40 | | 0.000 |
| Residual Error | 12 | | | 0.432 | | 0.432 | | 0.036 | | - | | - |
| Lack-of-Fit | 10 | | | 0.432 | | 0.432 | | 0.043 | | 129643.66 | | 0.000 |
| Pure Error | 2 | | | 0.000 | | 0.000 | | 0.000 | | - | | - |
| Total | 26 | | | 788.664 | | - | | - | | - | | - |

*R^2^=* 99.95; CV =2.48916; Adj-*R^2^ =* 99.88*;* Pred-*R^2^ =* 99.68; SS- sum of squares; DF- degree of freedom; MS- mean square.

*^a^ The observed values of protease production were the mean values of duplicates*

Non-significant at *P*<0.10.

**Table S3 A. Effect of various carbon and nitrogen sources on production of protease from *Streptomyces* sp. MAB18.**

| **Carbon sources** | **Enzyme production**  **(U/mL)** | | | **Nitrogen sources** | | **Enzyme production**  **(U/mL)** | |
| --- | --- | --- | --- | --- | --- | --- | --- |
| Control | 100 | | | Control | | 100 | |
| Glucose | 165±0.85 | | | Peptone | | 121.8±0.76 | |
| Maltose | 145.03±0.72 | | | Yeast Extract | | 133.87±0.71 | |
| Lactose | 52.73±0.66 | | | Beef extract | | 84.6±0.30 | |
| Starch | 93.37±1.19 | | | Malt extract | | 34.4±0.79 | |
| Fructose | 83.30±0.81 | | | Soybean meal | | 123.8±1.04 | |
| Arabinose | 56.03±1.15 | | | Casein | | 156.8±1.25 | |
| Sucrose | 123.57±0.64 | | |  | |  | |
| Xylose | 29.17±0.45 | | |  | |  | |
| Raffinose | 24.43±0.86 | | |  | |  | |
| **(B) Summary of purification steps of protease from *Streptomyces* sp. MAB18.** | | | | | | | |
| **Purification steps** | **Volume (mL)** | **Total activity (U/mL)** | **Total protein (mg)** | | **Specific activity (U/mg)** | **Purification (fold)** | **Recovery (%)** |
| Crude extract | 100 | 8262 | 123.20 | | 67.06 | 0.0 | 100 |
| (NH_4_)_2_SO_4_ precipitate | 45 | 3552 | 42.48 | | 83.62 | 1.25 | 42.99 |
| DEAE-Cellulose | 25 | 1766 | 8.30 | | 212.77 | 2.54 | 39.02 |
| Sephadex G-50 | 5 | 384 | 0.16 | | 2398.36 | 17.13 | 21.62 |

The values are mean ± SE, n = 3

**Table S4 Effect of metal ions and chemicals on activity of the purified protease from *Streptomyces* sp. MAB18**

| **Alkaline protease activity (%)** | | | | | | |
| --- | --- | --- | --- | --- | --- | --- |
| **Metal** | **Concentration (mM)** | | | | | |
|  | **1** | **3** | | **5** | | **10** |
| Control | 100 | 100 | | 100 | | 100 |
| FeCl_2_ | 121.27±0.58 | 126.60±0.89 | | 132.27±0.58 | | 144.27±0.61 |
| HgCl_2_ | 14.40±0.62 | 18.73±0.41 | | 21.07±0.48 | | 21.73±0.58 |
| MgCl_2_ | 116.73±0.52 | 119.73±0.52 | | 124.73±0.64 | | 135.73±0.58 |
| AgNO_3_ | 73.03±1.18 | 74.70±0.64 | | 82.70±0.53 | | 88.37±0.24 |
| CuCl_2_ | 53±0.44 | 59.33±0.32 | | 60.67±0.58 | | 65.33±0.52 |
| PbCl_2_ | 14.40±0.64 | 18.40±0.61 | | 19.73±0.72 | | 21.40±0.61 |
| CaCl_2_ | 72.13±0.93 | 75.47±0.44 | | 78.47±0.73 | | 85.47±0.73 |
| NiCl_2_ | 49.57±0.41 | 51.90±0.72 | | 52.57±0.75 | | 55.57±0.66 |
| MnCl_2_ | 78.83±0.35 | 80.17±044 | | 81.50±0.64 | | 95.50±0.64 |
| **Chemicals** | | | **Concentration** | | **Mannanase activity (%)** | |
| **Control** | | | - | | 100 | |
| SDS | | | 0.1^a^ | | 23±0.58 | |
| Triton X-100 | | | 0.5^b^ | | 123.33±0.88 | |
| Tween-80 | | | 0.1^b^ | | 85±0.58 | |
| 1,10- phenanthroline | | | 5 mM | | 34±1.15 | |
| DMSO (Dimethyl sulfoxide) | | | 1^b^ | | 114.33±0.88 | |
| EDTA | | | 5 mM | | 37±0.58 | |
| Sodium sulphite | | | 0.1^a^ | | 114±0.58 | |
| Isopropanol | | | 1^b^ | | 76±058 | |
| β-mercaptoethanol | | | 0.1 mM | | 105±0.58 | |

*^a^ w/v*

*^b^ v/v*

The values are mean ± SE, n = 3
